# Supplementary material for: Apoptotic Effects of Drug Targeting Conjugates Containing Different GnRH Analogs on Colon Carcinoma Cells
Source: Int J Mol Sci. 2019 Sep 8;20(18):4421. doi: 10.3390/ijms20184421 (PMC6769516; doi:10.3390/ijms20184421)
Supplement: Supplementary file 1 [file ijms-20-04421-s001.pdf]

International Journal of
Molecular Sciences

MDPI

Supplementary Material

Apoptotic effects of drug targeting conjugates containing different GnRH analogs on colon carcinoma cells

Eszter Lajkó, Rózsa Hegedüs, Gábor Mező and László Kőhidai

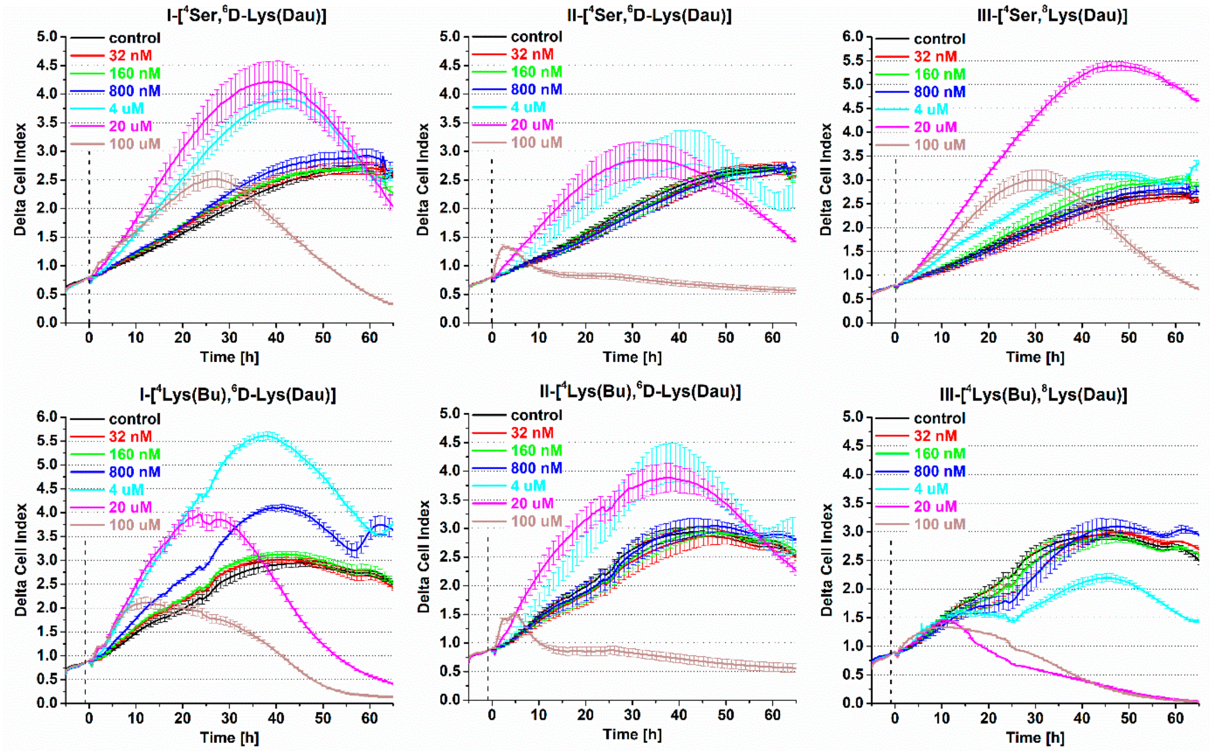

Figure S1. Time course study of the effect of Dau-GnRH-[4Ser/4Lys(Bu)] conjugates on HT29 cell viability by xCELLigence system

The Delta CI (Delta Cell index) refers to the difference of the CI value at the point in time of cell inoculation and the CI value at another given point in time.

Data shown in the figures represent mathematical averages of three parallels and ±SD values.

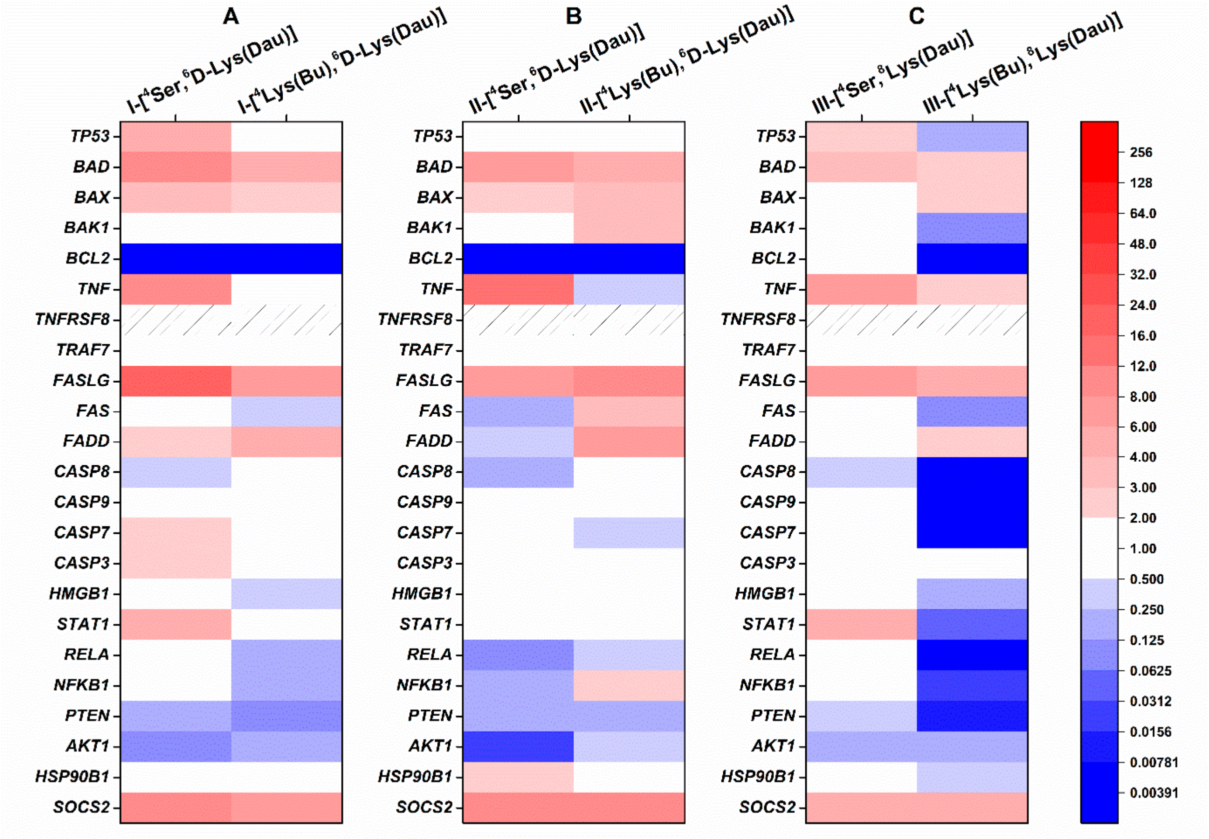

Figure S2 Comparison of the expression of human apoptosis-related genes in HT-29 cells treated with GnRH-[4Ser] and GnRH-[4Lys(Bu)] conjugates for 48 h. Effects of the conjugates pairs of GnRH-I (A), GnRH-II (B), GnRH-III (C) on gene expression were analyzed by a human apoptosis gene PCR array (RealTime ready Custom panel). Colors of heatmap showed significant fold changes in gene expression compared to control. Fold changes ≥ 2 and $p < 0.05$ were considered as significant. Hashed zone means invalid PCR results.

TP53: tumor protein p53 data; *BAD*: BCL2-associated agonist of cell death; *BAX*: BCL2-associated X protein; *BAK1*: BCL2-antagonist/killer 1; *BCL2*: B-cell CLL/lymphoma 2; *TNF*: TNF-alpha, Tumor necrosis factor ligand superfamily member 2; *TNFRSF8*: Tumor necrosis factor receptor superfamily, member 8; *TRAF7*: TNF receptor-associated factor 7; *FASLG*: Fas ligand, TNF superfamily member 6 (*TNFSF6*); *FAS*: TNF receptor superfamily member 6 (*TNFRSF6*); *FADD*: Fas (*TNFRSF6*)-associated via death domain; *CASP7*: caspase 7; *CASP3*: caspase 3; *CASP9*: caspase 9; *CASP8*: caspase 8; *HMGB1*: high-mobility group box 1; *NFKB1*: nuclear factor of kappa light polypeptide gene enhancer in B-cells 1; *RELA*: v-rel reticuloendotheliosis viral oncogene homolog A; *AKT1*: v-akt murine thymoma viral oncogene homolog 1; *PTEN*: phosphatase and tensin homolog; *STAT1*: signal transducer and activator of transcription 1; *SOCS2*: suppressor of cytokine signaling 2; *HSP90B1*: heat shock protein 90kDa beta (Grp94) member 1

Table S1 Efficacy of the Dau-GnRH-[^{4}Ser/^{4}Lys(Bu)] conjugates – Viability of HT-29 cells treated with the Dau-GnRH-[^{4}Ser/^{4}Lys(Bu)] conjugates at 10^{-4} M for 24, 48 and 72 h

Conjugates	Viability [1] (%) (control = 100 % ± 5.97-6,52)		
	**24 h**	**48 h**	**72 h**
I-[^{4}Ser,^{6}D-Lys(Dau)]	150.05 ± 8.55	43.82 ± 1.18	4.75 ± 1.59
II-[^{4}Ser,^{6}D-Lys(Dau)]	37.18 ± 3.08	16.91 ±1.91	20.43 ± 2.66
III-[^{4}Ser ,^{8}Lys(Dau)]	10.42 ± 11.48	68.63 ± 5.13	19.05 ± 0.97
I-[^{4}Lys(Bu),^{6}D-Lys(Dau)]	86.69 ± 4.84	15.50 ± 0.55	5.31 ± 1.27
II-[^{4}Lys(Bu),^{6}D-Lys(Dau)]	35.64 ± 3.41	19.40 ± 2.77	24.31 ± 4.20
III-[^{4}Lys(Bu),^{8}Lys(Dau)]	57.8 ± 0.80	12.52 ± 0.50	8.77 ± 0.61

[1] Each data represents the mathematical average of three parallels ± SD. The decrease in the Cell indices caused by the different treatments was normalized to the identical control and this value was given as 'Viability' in percent.
